# Pennsylvania Hospital

**Published:** 1838-01-31

**Authors:** 


### CLINICAL LECTURES.

### PENNSYLVANIA HOSPITAL.

[We regret that Dr. Coates' Lectures have been for some time past suspended, owing to a domestic calamity. Our next number will contain his Lecture, delivered on Wednesday, the 24th inst.]

FISTULA IN ANO.

*Wednesday, 10th Jan.* DR. RANDOLPH commenced:

I exhibit to you to day, gentleman, an individual, affected with a troublesome though not very unusual form of complaint, with the nature and treatment of which it is of the highest importance for you to be thoroughly familiar. This man is forty-three years of age, of a constitution naturally sound, and which he states has not been injured by intemperance, or excesses of any other description.

About four months ago, he was attacked with a phlegmonous tumour, situated on one side of the perinæum, near the anus, attended with considerable pain, swelling, and hardness. Extensive suppuration ensued, and the abcess finally burst externally, giving rise to a free discharge of matter, through the external opening thus formed. Since that time the sore has never healed, but has continued to discharge matter, through a fistulous sinus, formed near to the rectum, constituting the disease we term Fistula in Ano. This is the ordinary commencement of Fistula in Ano. It may likewise originate from erysipelatous inflammation attacking the nates: in this case the adjacent cellular texture may slough extensively, and give rise to the formation of cavernous ulcerations in the region of the anus, which may produce sinuses, and terminate in the formation of well marked fistulæ. When the inflammation and tumefaction are very considerable in the forming stage of the disorder, the pressure of the tumour upon the neck of the bladder and urethra may cause dysury or even total suppression of the urine.

Fistula in ano may present itself under several different forms. When the abscess opens externally only, and no communication has been established with the intestine, it is termed *incomplete* fistula. When there is a communication with the rectum, and an opening externally also upon the nates, it is termed a *complete* fistula; in this case, a probe, made to enter at the external orifice, may be carried along through the sinus into the rectum. A third variety of fistula is that in which there is an internal opening into the cavity of the rectum, without an external aperture. This is called *occult* or *blind* fistula. In the present instance, the man's own statement satisfactorily proves the existence of a complete fistula. He will tell you that from a short time after the formation of the external opening, he has been passing both wind and fœces through the sinus; this is conclusive evidence that it communicates with the rectum.

When the occurrence of inflammation is first noticed upon the nates, a prompt and vigorous application of antiphlologistic treatment may possibly bring about a resolution of the phlegmon, and the formation of a fistula may be prevented. Even after suppuration supervenes, if an early opening be made, and a free vent afforded for the discharge of matter, the abscess may sometimes heal as in any other part, without the creation of a sinus. But, unfortunately, the surgeon is usually not called in, until a fistulous sinus is completely formed, and then, in order for a cure to take place, a surgical operation is rendered indispensable. You may ask, why an abscess situated by the side of the rectum will not heal up, without surgical interference? and I will proceed to enumerate the causes which make such interference necessary. When the fistula is incomplete, if the external opening be small, large quantities of matter may accumulate within the cavity of the abscess, and by preventing the granulations, which are thrown out, from coming in contact with each other, render it impossible for the parietes of the abscess to unite, and the wound to heal up. The external orifice may likewise close up prematurely, and in this case the patient flatters himself that he is restored to health; but his hopes are soon disappointed, for the accumulation of matter is going on internally, and it finally makes its way out through the old opening, or forms new outlets for its egress. When the fistula is complete, at the time the patient goes to stool, his fœces are pressed into the cavity of the sinus, and passing through it, give him intolerable pain, and oppose an insuperable obstacle to a cure. When it is occult, the fœces may also collect within the cavity of the abscess, and, by distending the parts, occasion excruciating agony. When the abscess is situated upon one side of the sphincter ani muscle, upon every evacuation of the contents of the rectum, the action of this muscle draws the detached side of the intestine from its natural situation, and, by constantly breaking up any granulations that may have formed, effectually counteracts every effort of nature to a cure. After the Sinus has remained for a long time open, the parts become callous, and this condition constitutes more strictly a genuine Fistula in Ano, than either of the states which I have just described.

It is proper I should mention to you, that, occasionally very considerable obscurity attends the existence of these several varieties of fistula.

Both in the complete and incomplete form of the complaint, where it has continued for a great length of time, and where the inflammatory appearances have entirely subsided, it is very difficult even after a minute inspection of the parts, to decide positively as to the nature of the disorder.

In some instances, the external aperture closes up altogether for a short period, and at other times, it is so nearly closed, that nothing more is visible, than a small point or pimple perhaps, not larger than a pin's head. It sometimes happens also, that the sinus itself is so very tortuous, that it is exceedingly difficult to pass a probe down to its extremity, perhaps it will not enter for more than half an inch or an inch; under these circumstances you would scarcely suspect that the sinus extended itself by the side of the rectum. These appearances however, cannot be at all relied upon, inasmuch as the matter goes on accumulating, until it has either to force its way out through the old opening, or else to form new ones.

I was consulted not long since in the case of a gentleman, who had been labouring under an incomplete fistula, for the period of two years; in this case, the inflammatory symptoms had so entirely subsided, that upon a very minute examination, I could discover nothing but a small pimple, not larger than the head of a pin, unattended with the least discolouration. The medical gentleman, who had charge of this patient, could not believe that the sinus ran up along the side of the rectum, because he was unable to introduce a probe into it, to the distance of more than an inch. He endeavoured to close up the aperture by the application of lunar caustic and the solution of the sulphate of copper, &c., but finding it obstinately refuse to heal, he called me into consultation. I was of the opinion that the sinus extended itself along the side of the rectum, notwithstanding his inability to detect its course with the probe. This I was for a time unable to accomplish myself, but, by persevering in the introduction of probes of different sizes, and varying their direction, I finally succeeded in tracing the sinus up to the side of the rectum. The fistula was afterwards easily cured by the usual operation.

Considerable obscurity sometimes attends upon the formation of an occult fistula. Two or three years since, a gentleman from Ohio, came to this city, to place himself under my care, for the relief of an affection of this nature. For eight or ten months previous to this time, he had been troubled with a discharge of purulent matter per anum,—attended however with very little pain. He told me that he had never been afflicted with hemorrhoids, or any irritation of an unusual character in the neighbourhood of the rectum. This discharge distressed him exceedingly; though not painful, it entirely unfitted him for the enjoyment of social intercourse. He would feel the matter trickling down at intervals during the day, and would be obliged to retire privately, to cleanse the parts. I stated to him, that it was my opinion, he had occult fistula; and that the disease probably extended upward, for a considerable distance; and that it would, sooner or later, show itself externally. Two months afterwards, he began to suffer a greater degree of pain, in voiding his stools. Soon after this, an enlargement became apparent in the buttock, attended with redness, inflammation, and excruciating pain. Upon pressing upon the buttock, I was able to cause a discharge of pus from the anus. I now ordered mild and emollient applications to the part, such as fomentations and poultices, for the purpose of hastening the progress of the suppuration. Shortly afterwards, fluctuation became evident, and I opened the abscess. The disease had therefore been, in this case, near twelve months in forming. It extended to a considerable distance up along the rectum, and the external opening was distant three or four inches from the verge of the anus.

The treatment of fistula in ano must depend upon the particular state in which the disorder presents itself. Whenever you are called to a case, in which you find much inflammation and swelling in the neighbourhood of the anus, you should endeavour, by every possible means, to subdue the inflammation, and, by promoting resolution, prevent the occurrence of suppuration. For this purpose, you should have recourse to a strict antiphlogistic treatment: general bloodletting, if the case will admit of it, leeches around the inflamed part, low diet, and absolute rest, in the recumbent position. If the inflammation and swelling be so great as to press upon the neck of the bladder, or the urethra and produce strangury; to relieve this, you should resort to the warm bath, fomentations, and anodyne injections; and in some instances, the introduction of a catheter into the bladder to draw off the urine will be necessary. If the inflammation show any disposition to terminate in gangrene, Dr. Physick recommends a blister to be applied to the inflamed parts.

When suppuration is inevitable, open the abscess as early as possible, at the most prominent part, in order to prevent the extension of the disorder by ulceration. After you have opened the abscess, apply mild dressings to the wound, such as simple cerate, or mucilaginous poultices. The old surgeons used in these cases, to cram the sore with lint and other substances, to make it heal, as they said, from the bottom. The effect of this treatment would be the reverse of that intended: the lint, by keeping the granulations from coming in contact with each other, would prevent the abscess from healing.

Notwithstanding, as I have already stated, abscesses, formed in the vicinity of the rectum, may, in some rare instances, be cured without an operation, still in the great majority of cases, particularly, when the side of the intestine is separated and detached from the adjacent parts, it is necessary, in order for a cure to take place, to divide the parts so as to make one cavity of the gut and sinus. To effect this, two different operations have been proposed: the one consists in the passage of a ligature through the orifices of the fistula and the anus; by the other, the two cavities are converted into one with a narrow curved bistoury. I shall call your attention, first to the mode of operation with the knife. The patient is to be laid over a table, either in a leaning posture, or flat upon his back, with his legs elevated; an assistant separates the nates. The surgeon then oils the index finger of his left hand, and introduces it into the rectum; he then passes a curved bistoury down to the extremity of the sinus; if the fistula be complete it comes in contact with the end of the finger in the rectum. He then withdraws them both together, dividing the soft parts between the intestine and sinus. If the fistula be incomplete, it is necessary to carry the point of the bistoury through the soft parts into the rectum, until the knife meets the end of the finger, converting it by this process into a complete fistula, after which the operation is the same. After the incision has been made, a dossil of lint is to be introduced into the cut, to arrest the hemorrhage, and to prevent the immediate reunion of the parts. If several sinuses exist, they are to be all laid open in the same manner. It sometimes happens that the fistula opens internally into the rectum so low down, that a director can be passed through the sinus into the rectum and out at the anus; in this case the parts can be divided much more conveniently by running the bistoury along the groove of the director.

Such is the usual mode of performing the operation by the knife. The fistula however occasionally extends far up by the side of the rectum, in which case, to divide the parts with the knife might involve important blood-vessels. Here the ligature may be used with advantage; I prefer one made of silk to any other material, especially to the metallic wires, recommended by Dessault and others. A common eyed probe, armed with a ligature, is to be passed through the sinus into the rectum, and out by the anus, so as to include the parts below the internal orifice of the fistula. This is to be tightened from time to time, until the parts are finally divided by ulceration. The advantages attending the application of the ligature, are the following: first, hemorrhage is avoided; secondly, the patient is not necessarily confined to his bed, but can walk about and take sufficient exercise to better preserve his general health. Another advantage attending the use of the ligature, is, the formation of a large gaping wound is prevented, which in some instances is apt not to heal; when the disease is very extensive, and the operation is performed by means of the knife, the least motion in the parts causes friction, and separates the two flaps, the granulations are thereby torn asunder, and occasionally the cut surfaces never unite. This is especially the case, when the fistula opens into the perinæum, where the parts are not so solid. I will only add, that occasionally fistula in ano occurs in patients affected with phthisis pulmonalis. In such a case, it is improper to operate, for this reason: although you may heal the fistula, the patient will still die of the pectoral complaint; and it is possible that the operation might hasten his death.

[After these remarks Dr. Randolph proceeded to perform the operation, by means of the knife, on the man, introduced into the amphitheatre. The internal orifice of the fistula was situated low down, the external opening was in the perinæum, and the surrounding parts had sloughed away much more, Dr. R. remarked, than if it had been situated in the buttock, owing to the presence of a greater mass of cellular texture. The usual introduction of lint was made. No future occurrence of hemorrhage was to be expected.]

While on this subject, I may mention, said Dr. Randolph, another morbid condition of the rectum, dependent on the presence of little sacs or pouches, just within the anus, at the lower extremity of the rectum. Dr. Physick first called our attention to this affection. The symptoms are an intolerable irritation and itching in the parts, with sometimes a discharge of matter. The plan of relief, is, to hook down the pouches with a bent probe, and cut them out with a pair of scissors.

*January 29th.* The wound is now granulating kindly. For a week past, its edges have been daily touched with lunar caustic.

---

### HYDROCELE.

*Wednesday, January 17th*—At eleven o'clock Dr. Harris, whose tour of duty commenced this day, entered the amphitheatre, and remarked as follows:

Gentlemen, I propose, to-day, to operate on a case of hydrocele. This disease usually appears in three forms. In the first, the fluid is lodged in the cellular texture of the scrotum; in the second, it is contained in a cyst, connected with the spermatic cord; and, in the third, and most frequent, the serum is collected in the tunica vaginalis. The last variety is the one on which I propose to operate, and I will therefore offer a few remarks regarding its character and treatment.

This disease is usually confined to one side of the scrotum; the tumour is of a smooth, oblong appearance,—unattended with discolouration of the skin, commences at the lower part of the scrotum, and thence gradually extends up the cord until it reaches near the external abdominal ring. In form, it resembles the large bell-pear, and hence has been called pyriform. In the early stage, it is soft and fluctuating. It cannot be diminished by pressure or position. If the tunica vaginalis and cremaster are not thickened, and the fluid is clear, the scrotum in a strong light will exhibit a semi-transparent appearance. In order to discover this transparency, the room should be darkened, a lighted candle should be closely held to one side of the scrotum, the surgeon should grasp the posterior part of the swelling, so as to render its fore part as tense as possible, and, then looking at the tumour from the side opposite to the candle, a transparency is at once discovered.

The testicle is usually placed at the posterior, and about the middle part of the swelling. Pressure at that part affords the usual sensation produced by squeezing the testes, and the gland will be seen in this position. In some cases, however, the testicle becomes adherent to the anterior part of the tunica vaginalis, so that the serosity accumulates laterally to, and below the testicle. Unless great care is observed in this case the testis may be wounded by a plunge from the trocar.

The nature of the fluid contained in hydrocele resembles serum. It is of a straw colour, is capable of coagulation by heat, acid, port wine, and sulphate of zinc. The contents of the hydrocele are as already stated, of a transparent colour, yet, in other cases, it is as colourless as spring water, sometimes of a milky appearance, and again of a dark chocolate colour. Sometimes flocculi are found swimming in the serum; and Velpeau has seen fatty and cartilaginous substances flow out with the serum from the sac.

The usual quantity of fluid taken from a hydrocele is from six to eight ounces. It is stated, however, on the authority of Mr. Cline, that he drew off six quarts of fluid from the celebrated historian Gibbon. There is a variety of hydrocele, of which the following case forms an example. A gentleman from Tennessee requested me to place him under treatment for a large tumour of the scrotum. On examination I found that the tumour possessed all the characteristics of hydrocele of the tunica vaginalis, but, when the patient was in a horizontal position, the fluid for the most part escaped within the abdomen. It was therefore at once inferred, that it was a case of congenital hydrocele. After consultation with my friend Dr. Horner, it was determined to draw off the fluid, apply an inguinal truss so as to excite inflammation and adhesion between the sides of the sac which passes through the inguinal canal. A piece of gum elastic catheter was passed into the sac of the tunica vaginalis, which, while it conveyed off the fluid as fast as it was secreted, produced at the same time, the necessary inflammation to obliterate the cavity. After this end was achieved, we found that fluid had again collected within a circumscribed cavity of about five inches in diameter, above Poupart's ligament extending towards the iliac fossa.

Finding that this swelling became tense and elastic, and rapidly increased in magnitude, we determined to dissect cautiously down to the tumour, to attach the sides of the sac to the parietes of the abdomen by the interrupted suture, then to puncture it and draw off the water. The subsequent treatment precisely corresponded with that of the hydrocele contained within the scrotum. As in the former case, the catheter remained in the cavity until it was obliterated. To all appearance, the patient left the city in a state of perfect restoration to health.

This I believe is a rare form of the disease. I have been able to find but one case similar to it, in the records of surgery. This is to be found in *Les Leçons orales* of Dupuytren. This eminent surgeon denominates it *L'hydrocele en bissac.* Dupuytren does not detail the treatment of his case.

Hydrocele may be confounded with Hernia, with sarcocele, with varicocele, and with hæmatocele. Hydrocele may be distinguished from hernia, by a return of the hernial tumour into the abdomen, by the latter receiving an impulse from coughing, by the hernia descending through the abdominal ring; while hydrocele forms at the bottom of the scrotum and ascends.

These two diseases sometimes combine in the same individual, in which case the hydrocele is found anterior to the hernia.

Varicocele may be distinguished from hydrocele by directing the patient to place himself in a recumbent position, when the varicocele, with the aid of slight pressure recedes.

From sarcocele it may be distinguished by its smoothness of surface, by its freedom from nodosities, the absence of pain and sensibility.

Hæmatocele may be distinguished from hydrocele by its weight, its want of transparency, its obscure fluctuation, and its being suddenly formed after a blow on the scrotum.

The causes of hydrocele of the tunica vaginalis are not very well understood. It frequently attacks young, healthy and robust subjects, has no connection with dropsical habits, and is generally an affection entirely local. It is a complaint, quite common in warm climates, and is thought to arise from frictions of the scrotum, and abrasions produced by riding on horseback. It has been thought by Ramsden and others, to arise from chronic irritation of the urethra. The disease has been also caused by an ill contrived truss on the spermatic cord. I saw one case occasioned by an injury of the testicle, by a blow from the pummel of a saddle.

There are several methods of treating hydrocele. 1st. by absorption, 2nd. by the palliative, and 3d. the radical method.

In young children, the first method is commonly successful. The child is purged, the scrotum is suspended, and stimulating washes are freely applied to the surface of it, until more or less irritation is produced. I have often used in such cases the muriate of ammonia dissolved in vinegar. The tinctura lyttæ is used for this purpose by Sir A. Cooper. This treatment often causes an absorption of the fluid. Though this method commonly succeeds in children, it almost always proves unsuccessful in adults. I have the pleasure, however, to present a case to you which forms an exception to this rule. You observe that the tumour is absorbed, by the treatment adapted to children. The credit of this successful case, is due to my colleague Dr. Randolph.

The *palliative* treatment consists in discharging the fluid by means of a lancet or trocar. There are circumstances which warrant us, at times, to prefer this methed. As for instance, where the health of the individual is unable to endure the excitement, which the severer method would produce; or, when it is connected with diseased testicle; or, from the great distention and magnitude of the sac. In such a state, it has been thought advisable to allow it to contract, and afterwards, to resort to radical means, when the tumour is less in magnitude.

There are cases too, where it may be inconvenient for the patient to be at rest, which is necessary in the performance of the radical cure, and yet he is anxious to be relieved from the inconvenience of the swelling. There have been some rare cases, where the simple discharge of the fluid has been followed by a permanent cure.

This simple operation has been sometimes attended with grave consequences. It is stated by Sir A. Cooper, that an aged gentleman on whom he operated, died on the sixth day afterwards, of gangrene of the scrotum. Velpeau and Green have published analogous cases.

Various operations have been proposed to accomplish a radical cure of hydrocele. Among them may be numbered the potential and actual cauteries, incisions, excision of a portion of the sac, setons, bougies, injections, and acupuncturation. The operations of cauterization, and excision of the sac, are now entirely abandoned. The manner of performing, and the relative value of each of the other operations, shall be briefly noticed.

The practice of incision originated with Celsus. The patient is placed in a semi-recumbent position, the surgeon seizes the tumour by its posterior face, by which the fluid is pressed forward, into the most prominent part of which, the bistoury is plunged. The director is now passed to the inferior part of the sac, and, with the bistoury, it is freely laid open. The cavity is filled with lint or flour, until suppuration is freely established. By such means, the cavity is obliterated by granulations. In a case of hydrocele, which was marked by some obscurity, I recently operated by incision, which proved successful, and without an unpleasant symptom. The tediousness of this curative process constitutes an objection to it; still it has warm advocates in Rush and Gama, who prefer it to all others.

The operation with the Seton has been attributed by Mr. S. Cooper to Franco. It has had advocates, in all countries.

The old method of performing this operation, was to pierce the sac by means of a trocar. After a portion of the fluid is withdrawn, a seton canula is to be forced through the trocar, until it points near the superior part of the tumour. A sharp pointed eyed probe, armed with a seton, is to be conveyed through the canula, and brought through the sac and integuments. The canulas are now withdrawn, and the seton allowed to remain until the necessary degree of inflammation is produced. Sir A. Cooper is in the habit of penetrating the hydrocele transversely with a curved needle and single thread, so as to include about an inch and a half of integument, and one inch of tunica vaginalis. The thread is then tied, and left to hang loosely in the cavity. In about a week, the part swells and reddens, when the seton should be withdrawn. The adhesive inflammation produces the cure.

The tent or bougie has been successfully used to excite adhesive inflammation in the tunica vaginalis. This is the favourite method of Baron Larrey. S. Cooper gives it as his opinion that the bougie produces too much inflammation of the testis. In several instances in which I have used it, no unpleasant effect followed the operation.

It has been stated by Morand, that a young gentleman, affected with hydrocele, accidentally sat on a long needle which penetrated the scrotum and tunica vaginalis. There resulted from this accident an entire cure of the disease in eight days, although of three years standing.—*Acupuncturation* has become one of the radical cures for this disease. Doctor Lewis used this method in fifty cases, and in not one instance did a failure take place.

I have had no experience in this operation, excepting in one case. In this, the operation entirely failed, though the punctures were at distant intervals. On each occasion, however, the fluid was entirely evacuated, without giving rise to either pain or subsequent inflammation. It is certainly an admirable method to evacuate the contents of the sac.

The operation by *injections* has been long considered one of the most effectual remedies for hydrocele. It first originated with Celsus—revived by a military surgeon of the name of Monro, and afterwards, became the favourite operation of Sir James Earle. It has been also the favourite remedy of the surgeons of this country.

The instruments requisite to perform this operation, consist of a trocar and canula, and a syringe, the pipe of which is well fitted to the mouth of the canula. The fluid to be injected is composed of equal parts of Port wine and water—ʒss of sulphate Zinc to f℥viii of water—one part of spirits of wine to five of water—or even pure water has been sometimes used. The favourite fluid now injected is diluted tincture of Iodine. This article has been used by Velpeau in thirty-eight cases successfully. This injection is most certain in its effects, accomplishes a cure in a shorter period, gives rise to less pain, and has not been in any instance attended with unpleasant effects. Even if by accident this injection has been thrown into the cellular texture of the scrotum, no active inflammation supervenes. If the diluted Port wine is thrown into this texture, the whole scrotum has been known to slough. Such is the experience of Velpeau with iodine injections.

I have not yet used this fluid in the treatment of hydrocele, but propose to do so, with this patient. [Dr. Harris now drew off the contents of the hydrocele, in the manner already indicated, but finding the testicle diseased, he determined not to use the injection, until a subsequent period. In the meantime, he stated the patient would be placed under treatment for sarcocele.]

